# Malposition of an Internal Aortic Annuloplasty Ring After Acute Type A Aortic Dissection Repair

**DOI:** 10.7759/cureus.66061

**Published:** 2024-08-03

**Authors:** Chikashi Nakai, Eduardo Danduch, Saeed Tarabichi, Sanjay Samy

**Affiliations:** 1 Cardiothoracic Surgery, Albany Medical Center, Albany, USA

**Keywords:** acute type a aortic dissection, modified florida sleeve, aortic annuloplasty, aortic dissection, redo aortic valve replacement

## Abstract

Poor tissue quality of adventitia and intima makes aortic root repair complex in patients with acute type A aortic dissection. The management of aortic root repair remains controversial. Internal aortic annuloplasty devices provide a standardized aortic valve repair technique to reduce and prevent annular dilation, while the modified Florida sleeve (mFS) procedure is an aortic root remodeling technique that does not require coronary artery reimplantation. However, little is known about the long-term durability of internal aortic annuloplasty with the hemispheric aortic annuloplasty remodeling ring (HARRT) combined with a mFS procedure in acute type A aortic dissection repair. A 52-year-old man had initial type A aortic dissection repair with an internal aortic annuloplasty ring and a mFS technique. He presented with dyspnea on exertion and intermittent chest pain one year later. Transesophageal echocardiogram revealed malposition of aortic annuloplasty ring and severe aortic insufficiency. He underwent a redo sternotomy with aortic valve replacement. Intraoperative findings demonstrated the aortic annuloplasty ring had dislodged from under the left and right coronary annulus and was adherent to the base of the noncoronary leaflet. The annuloplasty ring and aortic valve leaflets were excised and replaced with a mechanical aortic valve.

## Introduction

The repair of the aortic root and aortic valve in patients with acute type A aortic dissection (ATAAD) is important; however, the management has not been established because of technical complexity [1]. Aortic annuloplasty with an internal hemispheric aortic annuloplasty remodeling ring (HARRT) 300 ring provides a standardized aortic valve repair technique for aortic insufficiency (AI) to reduce and prevent annular dilation [2,3]. Aortic ring annuloplasty could have technical problems in the first clinical trials, but results improved further with more experience [4]. Aortic ring annuloplasty is currently considered an essential component of aortic valve repair and valve-sparing root surgery [5]. Aortic root remodeling with external ring annuloplasty enabled avoidance of both ventriculoarotic junction and recurrent AI in young patients with ATAAD [6]. The modified Florida sleeve (mFS) procedure is an aortic root remodeling technique that does not require coronary artery reimplantation and is the alternative option instead of the Bentall procedure [7]. Little is known about the long-term outcome of internal aortic annuloplasty with the HARRT300 ring combined with the mFS procedure for ATAAD. This is a case report on the durability and long-term outcome of an internal aortic annuloplasty ring with mFS in a patient with ATAAD.

## Case presentation

**Figure 1:**
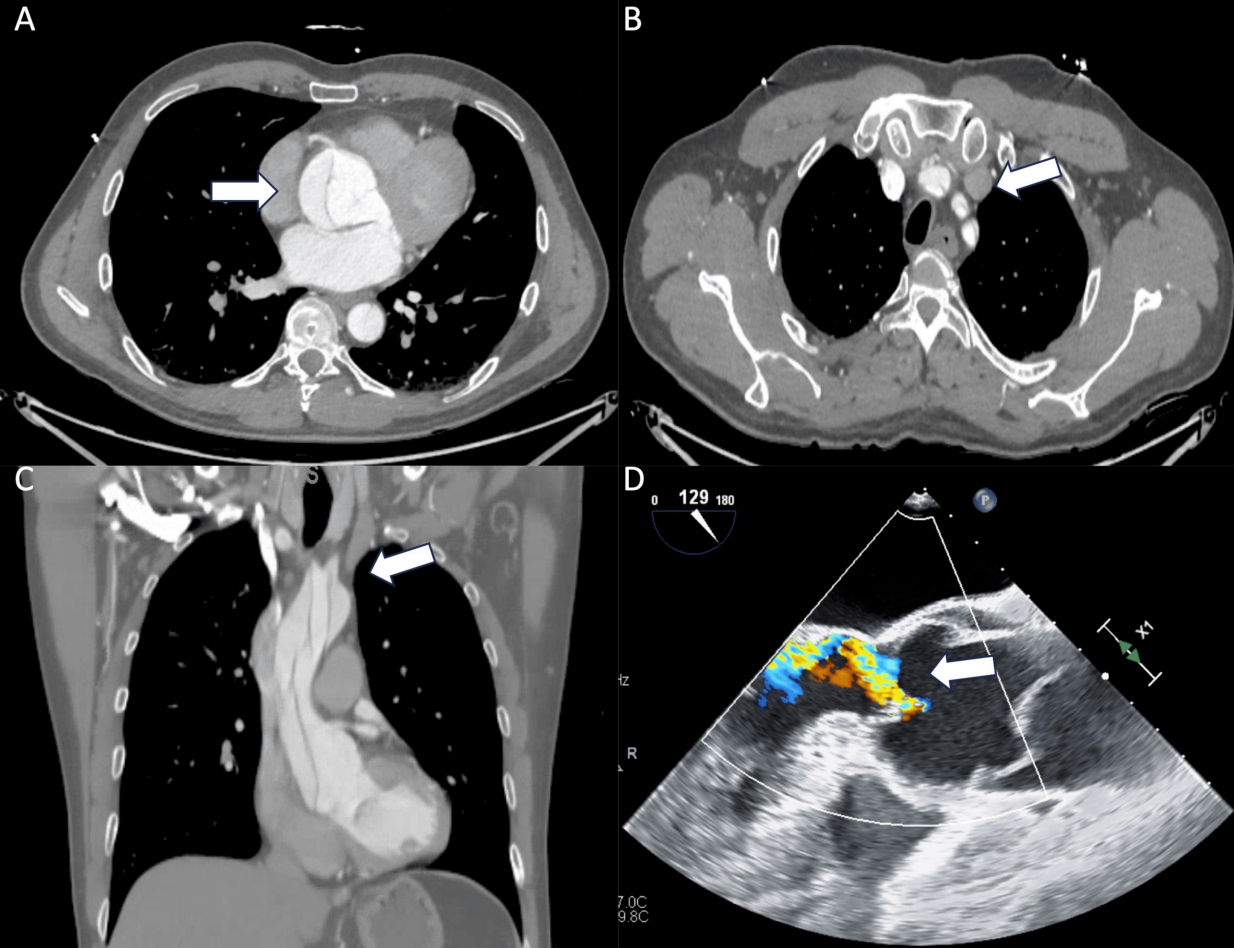
Preoperative chest computed tomography with contrast (A) Aortic dissection arising from the aortic root (white arrow). The aortic root is 6.2 x 5.0 cm in diameter (B, C). Aortic dissection extended into the innominate artery, the left carotid artery, and the left subclavian artery (white arrow). (D) Transesophageal echocardiogram: moderate aortic insufficiency (white arrow)

**Figure 2:**
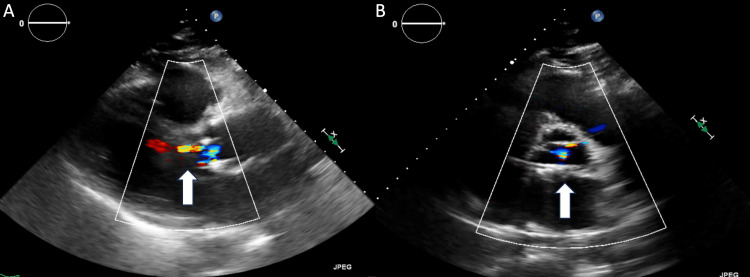
Transthoracic echocardiogram on postoperative day eight (A, B) Left ventricular ejection fraction > 55%, mild aortic insufficiency (white arrow)

**Figure 3:**
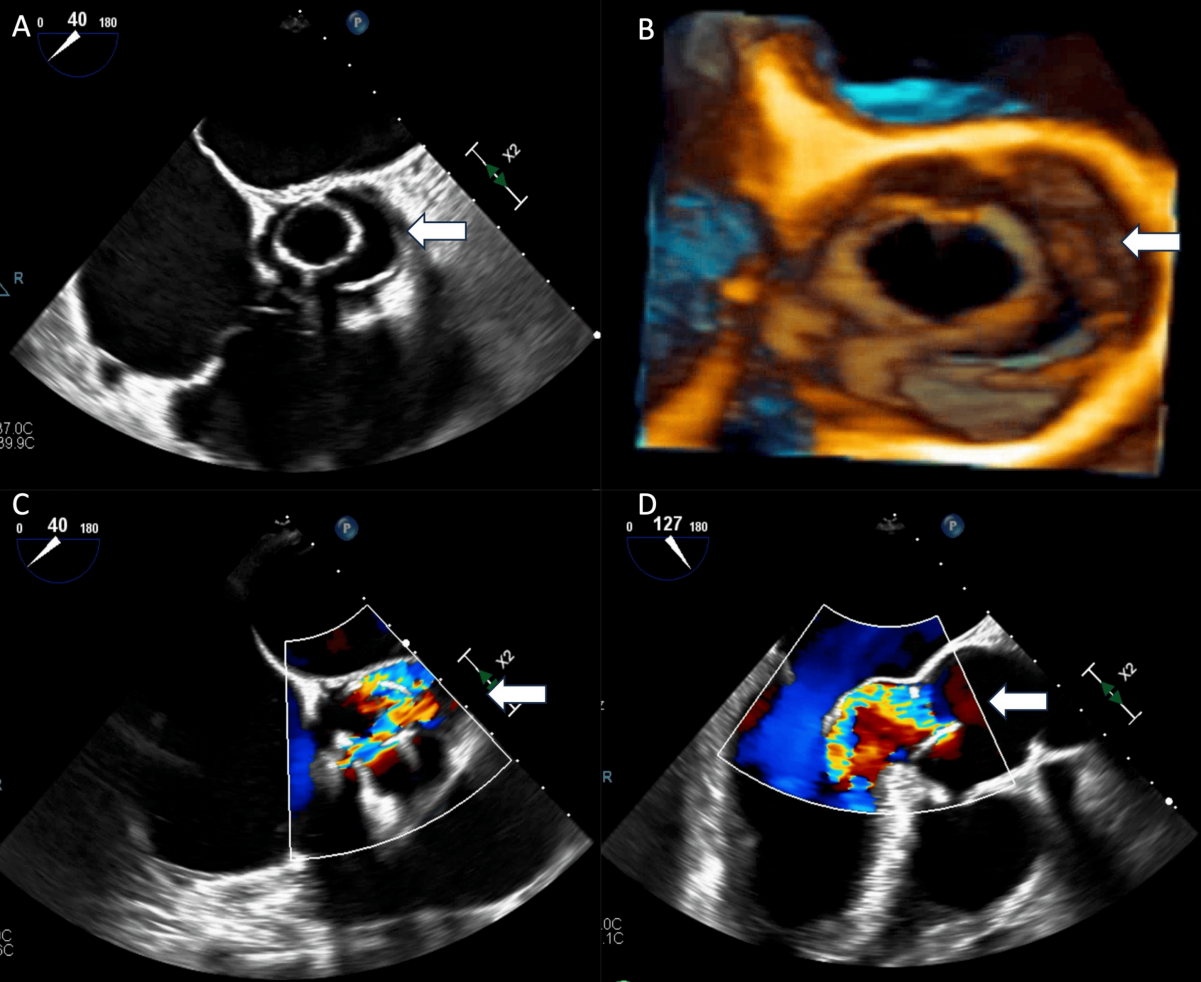
Transesophageal echocardiogram on postoperative five months (A, B) Aortic annular ring not attached to the anterior aspect of the aortic root (white arrow). (C, D) Severe aortic insufficiency detected with eccentric jet (white arrow)

A 52-year-old man without a past medical history presented to our emergency department with a sudden onset of chest and back pain. Computer tomography angiography confirmed ATAAD arising from the aortic root, the aortic root 6.2 x 5.0 cm in diameter (Figure 1A), and aortic dissection extended into the innominate artery, the left carotid artery, and the left subclavian artery (Figures 1B, 1C). A transesophageal echocardiogram (TEE) revealed moderate AI (Figure 1D). He underwent standard sternotomy, artery canulation through the right femoral artery, and venous cannulation through the right atrium. Hemiarch replacement with a 30 mm Dacron graft was performed during deep hypothermia circulatory arrest. Due to infiltrative hematoma into the aortic root, and poor tissue quality, it was decided not to perform the Bentall procedure. Eight horizontal mattress sutures were placed approximately 5-7 mm below the aortic annulus and driven outside the aorta in a circular fashion after being placed through 21 mm HAART300 aortic annuloplasty ring at the commissures and around the ring along the annulus to stabilize the annulus. A 30 mm Dacron sleeve was placed around the aortic root, the mattress sutures were placed through the Dacron sleeve, and the sleeve was cut in two places to allow for the left coronary ostia and right coronary ostia (mFS procedure). Cardiopulmonary bypass time was 129 minutes, while aortic cross-clamp time was 93 minutes. There was no issue intraoperatively. Postoperative transthoracic echocardiogram revealed left ventricular ejection fraction > 55% with mild AI (Figures 2A, 2B). He was discharged to a rehabilitation facility on postoperative day (POD) 28 and subsequently went back home. His follow-up TEE five months after the initial ATAAD repair showed that the aortic annular ring was not attached to the anterior aspect of the aortic root (Figures 3A, 3B) complicated by severe AI with eccentric jet (Figures 3C, 3D). He started to complain about dyspnea on exertion and intermittent chest pain a year after the initial type A dissection repair. He was taken to the operating room for a redo sternotomy with aortic valve replacement. After the redo sternotomy, an 8 mm Dacron graft was sewn to the innominate artery for arterial cannulation, and venous cannulation was accessed through the right atrium. After the establishment of cardiopulmonary bypass, an aortic cross-class clamp was applied for the superior portion of the Dacron graft, and cardiac arrest was obtained with retrograde cardioplegia. Intraoperative findings were the aortic annuloplasty ring that had dislodged from under the left and right coronary annulus and was adherent to the base of the noncoronary leaflet. The annuloplasty ring and aortic valve leaflets were excised and replaced with a 31 mm St. Jude mechanical aortic valve in a supra-annular position. There were no issues perioperatively. He was discharged home on POD nine.

## Discussion

The patient who underwent ATAAD repair with an internal annuloplasty ring and mFS procedure required explantation of the ring with redo aortic valve replacement subsequently. To the best of our knowledge, limited data exist to place the internal annuloplasty ring for patients with ATAAD complicated by aortic root dilation. Papakonstantinou et al. reported that a geometric annuloplasty ring, HAART300, was a safe and effective approach to spare the autologous aortic valve in 20 patients with tri-leaflet AI in the short-term follow-up [2]. In a trial of internal aortic ring annuloplasty, valve-related complications were low over a three-year follow-up. However, a leaflet tear and partial ring dehiscence were documented because of surgical technical inaccuracies [8]. In our case, the annuloplasty ring might be too small, causing the dislodgement of the ring subsequently. Jawitz et al. [9] reported that 20 patients with AI underwent aortic valve repair with an internal annuloplasty ring and remodeling valve-sparing root replacement with selective sinus replacement. There was no perioperative mortality and no late annuloplasty-related complications at early median postoperative follow-up of 11 months [9]. Those studies were analyzed in patients with significant AI without type A aortic dissection. The Florida sleeve procedure simplified aortic root remodeling without coronary reimplantation, and it can be performed safely in patients with ATAAD. The early and long-term outcomes were comparable to other valve-sparing root replacements [10]. A neo-adventitia technique, one of mFS, showed great promise in patients with ATAAD to avoid uncontrollable bleeding from a proximal anastomotic line and prevention of future root dilatation [7]. In our case, the initial ATAAD repair with an internal aortic annuloplasty ring and mFS procedure was successful. However, the annuloplasty ring was dehisced from coronary sinuses subsequently. The malposition of the annuloplasty ring led to severe AI. This could be explained by the fact that the tissue around the aortic root was fragile in patients with ATAAD. Eventually, the patient required the redo aortic valve replacement with a mechanical aortic valve.

## Conclusions

The use of the combined mFs procedure with an internal aortic annuloplasty ring implantation may not provide long-term freedom from aortic insufficiency in patients with acute type A aortic dissection, although short-term outcomes were satisfactory with stable aortic annulus and regulated aortic insufficiency. This is the first report of the long-term outcome of internal aortic annuloplasty with the HARRT ring combined with the mFs procedure in acute type A aortic dissection repair.

## References

[1] Yang B, Norton EL, Hobbs R (2019). Short- and long-term outcomes of aortic root repair and replacement in patients undergoing acute type A aortic dissection repair: twenty-year experience. J Thorac Cardiovasc Surg.

[2] Papakonstantinou NA, Kogerakis N, Kantidakis G, Athanasopoulos G, Stavridis GT (2021). A modern approach to aortic valve insufficiency: aortic root restoration via HAART 300 internal annuloplasty ring. J Card Surg.

[3] Tabrizi NS, Stout P, Richvalsky T (2022). Aortic valve repair using HAART 300 geometric annuloplasty ring: a review and echocardiographic case series. J Cardiothorac Vasc Anesth.

[4] Gerdisch MW, Reece TB, Emerson D (2022). Early results of geometric ring annuloplasty for bicuspid aortic valve repair during aortic aneurysm surgery. JTCVS Tech.

[5] Youssefi P, El-Hamamsy I, Lansac E (2019). Rationale for aortic annuloplasty to standardise aortic valve repair. Ann Cardiothorac Surg.

[6] Kato Y, Sasaki K, Yamauchi H (2020). Aortic root remodelling with external ring annuloplasty in acute type A aortic dissection. Interact Cardiovasc Thorac Surg.

[7] Heo W, Min HK, Kang DK, Jun HJ, Hwang YH, Choi JH, Wi JH (2013). A modified root reinforcement technique for acute aortic dissection with a weakened aortic root: a modified Florida sleeve technique and two cases report. J Cardiothorac Surg.

[8] Mazzitelli D, Fischlein T, Rankin JS (2016). Geometric ring annuloplasty as an adjunct to aortic valve repair: clinical investigation of the HAART 300 device. Eur J Cardiothorac Surg.

[9] Jawitz OK, Raman V, Anand J (2020). Aortic valve repair with a newly approved geometric annuloplasty ring in patients undergoing proximal aortic repair: early results from a single-centre experience. Eur J Cardiothorac Surg.

[10] Alhussaini M, Jeng EI, Martin TD, Fillion A, Beaver TM, Arnaoutakis GJ (2022). Florida sleeve is a safe and effective technique for valve salvage in acute stanford type A aortic dissection. J Card Surg.

